# Health resource utilisation by patients with neuroendocrine tumours with or without carcinoid heart disease: a multinational study

**DOI:** 10.3332/ecancer.2020.1141

**Published:** 2020-11-13

**Authors:** Hugo Tanaka, Deise Uema, Juliana F M Rego, Rui F Weschenfelder, Nathalia D’Agustini, Duilio R Rocha Filho, Juan M O’Connor, Romina Luca, Jose Eduardo R Nuñez, Milton José de Barros e Silva, Rachel P Riechelmann

**Affiliations:** 1 Department of Clinical Oncology, AC Camargo Cancer Center, São Paulo 01509-900, Brazil; 2 Division of Health Care Sciences Center for Clinical Research and Management Education Dresden; 3 Hospital Universitário Onofre Lopes, Natal 59012-300, Brazil; 4 Hospital Moinhos de Vento, Porto Alegre 90035-001, Brazil; 5 Hospital Universitário Walter Cantídio, Fortaleza 60420-570, Brazil; 6 Hospital de Gastroenterología Bonorino Udaondo, C1264AAA Buenos Aires, Argentina; 7 Instituto do Câncer do Estado de São Paulo, São Paulo, Brazil

**Keywords:** neuroendocrine tumours, carcinoid heart, health resource, public health, Latin America

## Abstract

**Background:**

Carcinoid heart disease (CHD) is a rare and severe complication from carcinoid syndrome which may be associated with high health resource utilisation (HRU). We aimed to compare HRU between patients with and without CHD.

**Methods:**

Multicentre retrospective study of 137 consecutive patients with neuroendocrine tumours (NET) and elevated urinary 5-hydroxyindoleacetic acid treated in seven large hospitals in Latin America. We used the chi-squared test for binary variables and the Mann–Whitney test for quantitative correlations. Variables were entered into a multivariable linear regression model for higher HRU.

**Results:**

One-third of the patients had (45) had CHD. Patients with CHD had significantly more emergency visits and echocardiograms as compared to patients without CHD. In the bivariate models, CHD (R^2^ = 0.61, *p* = 0.01), private health system (*R*^2^ = 0.63, *p* = 0.02) and simultaneous cardiovascular comorbidities (*R*^2^ = 0.61, *p* = 0.04) were associated with a higher HRU. The multivariate model pointed out the accumulated effect of variables on HRU (*R*^2^ = 0.2, *p* < 0.01).

**Conclusions:**

NET patients with CHD present higher HRU independently of other clinical factors or health system. Effectively treating carcinoid syndrome, and likely delaying the onset of CHD, may potentially reduce the amount of HRU by these patients.

## Introduction

Carcinoid heart disease (CHD) is a rare and unique cardiac manifestation that occurs in patients with advanced neuroendocrine tumours (NETs) and carcinoid syndrome (CS) [1]. The exact mechanisms causing CHD are poorly understood, although chronic exposure to elevated serotonin serum levels is probably the main cause [2]. Responses to vasoactive substances such as serotonin lead to myofibroblast proliferation and local deposition of the extracellular matrix, with consequent development of matrix-rich fibrous endocardial plaques in right cardiac valves [3]. CHD represents a major cause of morbidity and mortality, as it evolves to right heart failure [1]. Therefore, international guidelines recommend the screening of CHD with an echocardiogram in patients with elevated urinary 5-hydroxyindoleacetic acid (5-HIAA), aiming at an early diagnosis [4, 5]. Once CHD is established, surgical valve replacement is the only potentially curative treatment.

CHD has been described in up to 34.5% of patients with both NETs and CS in Latin America [6]. Delayed diagnosis and limited access to effective therapies may negatively affect the lives of patients with CHD. Indeed, the odds of presenting CHD in Latin America were 4.76 times higher for those treated in the public setting, where availability of NET-directed therapies is limited, when compared to patients managed in a private setting [6]. Intuitively, because CHD is associated with high morbidity, it may also be linked to high health resource consumption, thus overloading health systems. Therefore, this study aimed to quantify the health resources utilised by NET patients according to the presence of CHD.

## Methods

This was a retrospective multicentre study which included consecutive NET patients with elevated 5-HIAA treated in seven large different hospitals in Latin America: AC Camargo Cancer Centre, São Paulo, Brazil; Instituto de Câncer do Estado de São Paulo, São Paulo, Brazil; Hospital Moinhos de Vento, Porto Alegre, Brazil; Hospital Universitário Walter Cantídio, Fortaleza, Brazil; Hospital Universitário Onofre Lopes, Natal, Brazil; Hospital de Gastroenterologia Bonorino Udaondo, Buenos Aires, Argentina; and Instituto Alexander Fleming, Buenos Aires, Argentina. The multicentric study was conducted in accordance with International Conference on Harmonisation—Guideline for Good Clinical Practice, guidelines and local laws, and the protocol was submitted and approved by local ethics committees. The sample used in this study was obtained from a database of 137 consecutive adult NET patients (19–85 years) with elevated urinary 5-HIAA to estimate the frequency and risk factors for CHD [6]. Due to the retrospective nature of this study, no formal CHD screening protocol was performed in the participating institutions; however, it is a common practice to screen patients with elevated levels of urinary 5-HIAA with echocardiograms annually or every 2 years. CHD was defined as at least moderate reflux/regurgitation in tricuspid valves. The study covered the period from January 2000 to July 2018. The median follow-up time was 39 months (range: 2.7–150.6 months) [6].

Demographic data, comorbidities, tumour characteristics, cancer treatments and information on heart conditions as well as relevant medical resources used by the patients were collected and organised from patients’ records by researchers at each centre as described by Uema *et al* [6]. Baseline characteristics were gathered from medical records at the time of carcinoid syndrome diagnosis (the date of elevated 5HIAA urinary test). Health resource utilisation (HRU), our primary endpoint, was defined based on the structure of these variables used by Casciano *et al* [7], This study compared resource use and practice patterns in advanced neuroendocrine tumours (NET) patients upon disease progression, between Germany, France, Brazil and Italy, which brings an international multicentric comparison of some assets used to treat patients with NETs. The HRU variables considered in this study were the number of elective medical visits, emergency visits, echocardiograms and computerised tomography (CT) scans; number and length of intensive care unit and/or hospital admissions; number of treatments with chemotherapy, hepatic embolisation and/or targeted therapies; and frequency of any somatostatin analogue therapy.

To quantify HRU, we compared the utilisation of each HRU variable between patients with and without CHD. The absolute frequency of each variable was calculated. Quantitative variables were assessed for normality based on histograms and the Shapiro–Wilk test. To compare qualitative variables between patients with or without CHD, the chi-square test was used, and for quantitative variables, we used the Mann–Whitney test. A multivariable linear regression model was performed to evaluate whether CHD was independently associated with HRU. The dependent variable, HRU, was considered as the total number of chemotherapy treatments, elective medical visits, emergency visits, echocardiograms, intensive care unit admissions and hepatic embolisations each patient underwent. The independent variables were age (continuous), gender, any cardiovascular comorbidity (coronary insufficiency, chronic arterial hypertension, cerebral vascular event, etc.), type of health system (private versus public) and presence of CHD. For that, Pearson’s correlation was used when the independent variable was quantitative, and analysis of variance (ANOVA) one-way when it was qualitative. For the multivariate model, we used the multiple regression.

All statistical tests were two-sided, with the *α* level set at 0.05. All analyses were case-complete, with denominators described.

## Results

Among 137 patients evaluated, 92 did not have CHD and 45 had a diagnosis of CHD. As shown in Table 1, patients with and without CHD presented, respectively, an average of 58 and 54 years, mean follow-up time of 49 and 42 months, 29 (64.44%) and 43 (46.74%) had concurrent cardiovascular comorbidities, 33 (73.33%) and 42 (45.65%) were female, and 34 (75.56%) and 47 (51.09%) were treated in a public health system. The clinical outcomes of patients with CHD have been published previously [6].

Table 2 shows HRU according to the presence of CHD. Patients with CHD had more elective medical appointments and echocardiograms as compared to patients without CHD. In addition, patients with CHD were treated more frequently with somatostatin analogues.

The linear regression model for HRU (Table 3) showed that the presence of CHD (*R*^2^ = 0.61, *p* = 0.01), being treated in a private healthcare system (*R*^2^ = 0.63, *p* < 0.01) and cardiovascular comorbidities (*R*^2^ = 0.61, *p* = 0.04) were independently associated with HRU. When the factors were evaluated together, we observed a joint influence of them on the resources used by the patients (*R*^2^ = 0.2, *p* < 0.01).

## Discussion

In this multicentre retrospective study, we evaluated HRU by patients with NETs, with or without CHD, to understand the health utilisation patterns of this clinical complication. We observed that patients with CHD had more elective medical appointments and echocardiograms and were treated more frequently with somatostatin analogues. We also found that CHD was independently associated with more HRU. To our knowledge, this is the first study addressing the HRU of CHD in a detailed manner.

Few studies have evaluated the costs of diseases/resources in the management of NET. An important cost analysis of older people in the USA with NET during their first year of diagnosis showed a significant association between carcinoid syndrome and the total costs, hospitalisations and outpatient costs [8]. Casciano *et al* [7] pointed out that advanced NET is associated with significant health resource use across subtypes and countries, and resource utilisation is likely to increase upon disease progression.

Patients with CHD tend to be sicker when compared with those without CHD because they end up developing serious and limiting symptoms from right-sided heart failure, which explains our results of higher HRU among these patients. Similar to our findings, a retrospective study that looked at the use and average cost of health resources among NET patients through the evaluation of medical claims of CHD (as surrogates of real CHD) of 406 insured US patients also observed that CHD patients experienced more HRU such as higher average number of hospitalisations (1.4 versus 0.7 days), office visits (22.8 versus 19.8) and outpatient services (22.3 versus 15.4), and longer hospital stays (4.3 versus 2.0 days; all *p* < 0.05) [9]. In our population, though, we did not find increased number of or length of hospitalisation among CHD patients. Such differences could be explained by our smaller sample and by distinct study populations. For example, CHD severity might have varied between study patients; while we included patients without symptoms with CHD diagnosis made only by echocardiogram, the North American study likely evaluated more severe CHD patients given that they used medical claims of CHD to classify patients as having CHD. In addition, the US study included only insured patients where access to supportive treatments and diagnostic tests is not as restricted as it happens in the public systems of Brazil and Argentina.

Patients with CHD may present symptoms only at the end of the disease process, and the clinical signs are difficult to obtain at the onset of CHD [10, 11]. Therefore, the diagnosis of CHD can be delayed if echocardiographic screening is not performed [12]. In the diagnosis and monitoring of NET, all patients with elevated urinary 5HIAA should be screened for the presence of CHD. There are guidelines that recommend the use of echocardiograms [13, 14] and may include other blood tests to complement the diagnosis [15, 16].

Given that the access to effective therapies to treat NET is suboptimal in Latin America, where NET patients with carcinoid syndrome treated in the public system have nearly the double incidence of CHD [6], the provision of efficacious treatments to NET patients could reduce the incidence of CHD and, consequently, the burden of HRU in public health systems of Latin American countries. For example, the Brazilian public system does not provide somatostatin analogues to treat NET, not even for functioning tumours. While the only reimbursed treatments for NET provided by the government are intravenous chemotherapy and alpha-interferon, some provinces, like Sao Paulo state, provides somatostatin analogues free of charge to functioning NET patients treated in their public institutions. However, access to targeted therapies for cancer is not homogeneous across the country.

Some limitations of our study include its retrospective nature, which may limit the validity of our findings, in addition to the rarity of the disease that makes it difficult to establish a large prospective cohort. Because our study was multinational, and nearly spanned a decade, we could not estimate the real costs associated with HRU in patients with carcinoid syndrome or whether CHD was linked to higher cost, as it was suggested by the US study [9]. Yet, our study brings clinically relevant data as it demonstrates HRU in a rare condition such as CHD. Another positive side of our study is that it included only patients with confirmed, rather than surrogate, diagnosis of CHD (defined as at least moderate right-valve dysfunction in echocardiograms). Finally, we have shown the feasibility of undertaking collaborative studies that address important issues for our region.

In conclusion, patients with CHD treated in Latin America present higher HRU, including more visits to emergency services and more elective echocardiograms. CHD was also associated with more HRU independently of other factors such as age, comorbid cardiovascular illnesses and health system. We encourage further work be done on the HRU and costs associated with NET and CHD, particularly evaluating the cost-effectiveness of NET-directed treatments in developing countries.

## Funding statement

This research was conducted as a part of the Neuroendocrine Tumours Fellowship Program sponsored by the Brazilian Society of Clinical Oncology in partnership with AC Camargo Cancer Center

## Conflicts of interest

Rachel Riechelmann has received honoraria and consultancy fees from Novartis and Ipsen. The other authors declare that they have no conflict of interest related to the publication of this manuscript.

## Figures and Tables

**Table 1.** Baseline demographic characteristics of the study population.

		With CHD (*n* = 45)	Without CHD (*n* = 92)	*p*-value
Age ± mean (years) and SD		58 ± 15	54 ± 12	0.07^a^
Follow-up time (months)		49 ± 37	42 ± 30	0.49^a^
Any cardiovascular comorbidity	With	64% (*n* = 29)	47% (*n* = 43)	0.05^b^*
	Without	36% (*n* = 16)	53% (*n* = 49)	
Gender	Female	73% (*n* = 33)	46% (*n* = 42)	<0.01^b^*
	Male	27% (*n* = 12)	54% (*n* = 50)	
Health system (relative frequency)	Public	76% (*n* = 34)	51% (*n* = 47)	0.01^b^*
	Private	24% (*n* = 11)	49% (*n* = 45)	

SD: standard deviation

a Mann–Whitney test

b Chi-square test

* *p* < 0.05.

**Table 2.** Comparative analysis of HRU among patients with or without CHD.

Resources	With CHD (*n* = 45)	Without CHD (*n* = 92)	*p*-value
**Quantitative (mean)**			
N of chemotherapy lines	0.2 ± 0.7	0.4 ± 1.1	0.53^a^
Elective medical visits	34 ± 32	25 ± 20	0.18^a^
Emergency visits	3 ± 3	2 ± 3	0.01^a^*
Echocardiograms	4 ± 2	2 ± 2	<0.01^a^ *
Intensive care unit admissions	0.4 ± 0.8	0.6 ± 1.1	0.38^a^
Hepatic embolization	0.7± 1.5	0.8 ± 1.5	0.80^a^
Total length of hospitalization	14 ± 17	18 ± 35	0.83^a^
N of CT scans^1^	8.3 ± 6.9	8 ± 6.3	0.88^a^
**Qualitative (relative frequency)**			
Treated with somatostatin analogue	96% (*n* = 43)	86% (*n*=79)	0.09^b^
Chemotherapy	14% (*n* = 6)	13% (*n*=13)	0.89^b^
Targeted therapies	27% (*n* = 12)	24% (*n*=22)	0.72^b^

CT: computed tomography)

a Mann–Whitney test

b Chi-square test

* *p* < 0.05

**Table 3.** Linear regression to evaluate factors associated with more HRU.

		Standardised regression coefficient	Standard error	Multiple Standard error	Adjusted *R*^2^	*p*-value	Multiple *p*-Value
Age	Age	−0.09	0.09		0.01	^a^0.35	
Gender	Female	−0.03	0.09		−0.01	^b^0.75	
	Male	0					
Cardiovascular comorbidities	With	0.18	0.09		0.61	^b^0.04^*^	
	Without	0					
Health system	Private	−0.27	0.09		0.63	^b^<0.01^*^	
	Public	0					
CHD	With	0.17	0.09		0.61	^b^0.01^*^	
	Without	0					
Cardiovascular * comorbidities * health system * CHS	Age	−0.17	0.09	26.86	0.20	^c^0.06	^c^<0.01*
	Gender	0.07	0.09			^c^0.45	
	Cardiovascular comorbidities	0.16	0.09			^c^0.07	
	Health system	−0.30	0.09			^c^<0.01*	
	CHD	0.23	0.09			^c^0.01*	

a Pearson correlation test

b ANOVA one-way test

c Multiple regression

* *p* < 0.05
